# Inhibiting PP2Ac*α* Promotes the Malignant Phenotype of Gastric Cancer Cells through the ATM/METTL3 Axis

**DOI:** 10.1155/2021/1015293

**Published:** 2021-08-24

**Authors:** Zhaoxiang Cheng, Shan Gao, Xiaojie Liang, Chao Lian, Jianquan Chen, Chao Fang

**Affiliations:** ^1^Department of General Surgery, The Affiliated Jiangning Hospital of Nanjing Medical University, Nanjing 211100, China; ^2^Department of Central Laboratory, The Affiliated Jiangning Hospital of Nanjing Medical University, Nanjing 211100, China

## Abstract

This article is aimed at exploring the relationship between the phosphatase 2A catalytic subunit C*α* (PP2Ac*α*, encoded by *PPP2CA*) and methyltransferase-like 3 (METTL3) in the malignant progression of gastric cancer (GC). Through analyzing the bioinformatics database and clinical tissue immunohistochemistry results, we found that abnormal PP2Ac*α* and METTL3 levels were closely related to the malignant progression of GC. To explore the internal connection between PP2Ac*α* and METTL3 in the progression of GC, we carried out cellular and molecular experiments and finally proved that PP2Ac*α* inhibition can upregulate METTL3 levels by activating ATM activity, thereby promoting the malignant progression of GC.

## 1. Introduction

The number of new gastric cancer (GC) cases exceeds 950,000 annually, with 78,300 deaths each year [1]. It is the fifth most common cancer in the world and the third leading cause of death due to cancer, accounting for 7% of the total number of cancer cases and 9% of cancer-related deaths [2]. Although the current D2 radical surgery for GC is quite mature and chemotherapy regimens are improving, the 5-year survival rate for advanced GC is only 20% [3]. The low survival rate of GC is closely related to the malignant phenotype of GC cells, which is characterized by proliferation, invasion, and distant migration. In recent years, targeted drugs that inhibit the malignant phenotype of GC cells, such as trastuzumab, have had a definite effect on HER-2-positive GC and can significantly prolong the survival of patients [4]. A phase II clinical study showed that rituximab can improve the prognosis of GC patients with high EGFR expression [5]. Ramulumab, which is a monoclonal antibody that targets VEGFR2, has a good antitumor effect, and its combination with paclitaxel for second-line treatment of advanced GC has been approved in some countries [6]. These cases suggest that molecular targeted drugs have the ability to improve the prognosis of GC patients. Therefore, further exploration of the molecular mechanism of the malignant phenotype of GC cells has important clinical significance for promoting GC therapy. In this study, two of the most common in vivo modifications (phosphorylation and methylation) were used as the entry point to study the molecular mechanism of GC.

Phosphorylation is an important biological event that regulates protein activity and stability, and it is of great significance for maintaining cells' physiological activities [7]. The steady-state phosphorylation of body proteins is mainly regulated by various phosphatases and kinases. Protein phosphatase 2A (PP2A), which is one of the main serine-threonine phosphatases in mammalian cells, maintains cell homoeostasis by counteracting most kinase-driven intracellular signaling pathways [8]. The PP2A catalytic subunit (which mainly refers to PP2Ac*α*) is the core basis of PP2A, and PP2Ac*α* dysfunction plays an important role in the occurrence and metastasis of some tumors. For example, immunohistochemistry and bioinformatics analysis showed that low expression of *PPP2CA* is closely related to colon cancer progression and poor prognosis; miR-650 promotes the malignant phenotype of undifferentiated thyroid cancer cells by inhibiting the expression of *PPP2CA*; upregulating the expression of *PPP2CA* can reverse the epithelial-mesenchymal transition, proliferation, and distant metastasis of prostate cancer. However, there have been no reports of PP2Ac*α* in GC.

In addition to protein phosphorylation, RNA methylation is indispensable for the maintenance of cell life activities, and the abnormal function of RNA methylation can lead to the occurrence of many diseases. N6-methyladenosine (m6A) modification is the most important and conservative RNA modification in cells [9]. Methyltransferase-like 3 (METTL3), as the core of the m6A-related methyltransferase complex, which plays an important role in the progression of various malignant tumors, for instances, upregulation of METTL3 promotes the proliferation of bladder cancer by accelerating the maturation of pri-miR221/222, promotes breast cancer progression by targeting Bcl-2, promotes the chemical and radio resistance of pancreatic cancer cells, and promotes the proliferation of colon cancer cells by inhibiting SOCS2. METTL3 also has been shown to play an important role in the occurrence and development of GC in recent years, but there is a lack of exploration of the regulatory factors of METTL3 in GC [10–14].

Previous studies have found that PP2A can inhibit the *MYC* gene and the AKT, KRAS, and NF-*κ*B proteins [15–18], as well as other oncogenes and tumor signaling pathways, thereby exerting a tumor suppressor effect. METTL3 tends to upregulate these oncogenes and signaling pathways [19, 20]. And in this study, through immunohistochemistry on gastric cancer tissue samples of 17 patients, it was found that in gastric cancer tissues, the positive rate of PP2Ac*α* was 2/17 (11.8%), and the positive rate of METTL3 was 14/17 (82.4%). As a result, the roles of PP2Ac*α* and METTL3 in malignant tumors may be very different, but there has been no research on the correlations between them when it comes to GC. Therefore, this study focused on exploring the interaction between PP2Ac*α* and METTL3 in the progression of GC.

## 2. Materials and Methods

### 2.1. Immunohistochemistry

GC pathological tissue wax blocks were provided by the Department of Pathology, the Affiliated Jiangning Hospital of Nanjing Medical University. Ten wax blocks with GC of pathological stage III-IV were screened out, and immunohistochemical sections were made from these tissue wax blocks. Anti-PP2Ac*α* antibody (Santa Cruz Biotechnology, Dallas, TX, USA) and anti-METTL3 antibody (Proteintech Group, Chicago, IL, USA) diluted to 1 : 300 were used for immunostaining. Based on the percentage of positive cells, 2 pathologists who did not know the clinical information independently evaluated the PP2Ac*α* and METTL3 staining intensities. The results were divided into the following categories: negative (-): 0 points, <25%; weakly positive (+): 1 point, ≥25, <50%; moderately positive (++): 2 points, ≥50, <75%; and strongly positive (+++): 3 points, ≥75%.

### 2.2. Cell Culture

Two human GC cell lines were used: MGC803 and BGC823 (Beyotime Biotechnology, Shanghai, China). The cells were cultured in Dulbecco's modified Eagle's medium (DMEM, HyClone, Inc., Logan, Utah, USA) added with 10% fetal bovine serum (FBS, Gibco, California, USA) and 1% antibiotics (penicillin/streptomycin, Gibco, California, USA). Cells were grown in a 5% CO_2_ incubator at 37°C.

### 2.3. Lentivirus-Mediated shRNA-Transfected Cells

According to the multiplicity of infection values of BGC823 and MGC803 (100 for both), GC cells were transfected with lentivirus-mediated sh-*PPP2CA* and sh-NC (Genomeditech Co., Ltd., Shanghai, China). The cells were harvested 48 hours after transfection. Then, puromycin was used to screen out positively expressing cells.

The shRNA sequences used were sh-*PPP2CA*-1, 5′-GATCCGTGGAACTTGACGATACTCTAACTCGAGTTAGAGTATCGTCAAGTTCCATTTTTT-3′; sh-*PPP2CA*-2, 5′-GATCCGCAGATCTTCTGTCTACATGGTTCAAGAGACCATGTAGACAGAAGATCTGCTTTTTTG-3′; and sh-*PPP2CA*-3, 5′-GATCCGGCAAATCACCAGATACAAATTTCAAGAGAATTGTATCTGGTGATTTGCCTTTTTTG-3′.

### 2.4. Clone Formation Experiment

Stably transfected gastric cancer cells were inoculated into a 6-well plate at a density of 1000 cells per well and cultured for 2-3 weeks. The medium was discarded and washed twice with PBS, and then, the cell clusters formed were fixed with 4% paraformaldehyde for 15 minutes and finally stained with crystal violet for 20 minutes. The image of cell clusters was taken, and the number of cell clusters was counted with ImageJ 1.8.0 software. All experiments were carried out in triplicate and repeated at least three times.

### 2.5. Cell Counting Kit-8 Experiment

Transfected GC cells were seeded into 96-well culture plates at a density of 1000 cells per well, and then at 24, 48, 72, and 96 hours of culture, cells were incubated with a 10 *μ*L Cell Counting Kit-8 (CCK8) reagent (Dojindo Molecular Technologies, Inc., Kumamoto, Japan) at 37°C for 2 h. The absorbance of each well was then measured in an automatic enzyme label meter (450 nm spectrophotometry).

### 2.6. 5-Ethynyl-2′-deoxyuridine Incorporation Test

Stably transfected GC cells were seeded into a 6-well plate at a density of 1.5 × 10^5^ cells per well for overnight culture; then, the cells were incubated with 10 *μ*M 5-ethynyl-2′-deoxyuridine (EdU) (Beyotime Biotechnology, Shanghai, China) in an incubator for 2 hours. The cells were then fixed, washed, permeabilized, and stained. Finally, the cells were observed with an inverted fluorescence microscope, and images were captured.

### 2.7. Scratch Test

The GC cell suspension was configured, and each cell suspension group was diluted to 5 × 10^5^ cells/mL. The above cell suspension was added to a double-well chamber (ibidi GmbH, Martinsried, Germany). Then, 70 *μ*L of cell suspension was added to each well of the chamber. The chamber was removed with sterile forceps after the cells adhered to the wall, and 2 mL of complete culture base was added. At the time points of 0, 12, and 24 hours, the cells were observed, and images were captured by using an inverted microscope equipped with a camera.

### 2.8. Transwell Experiment

A 24-well spreading gel invasion chamber (pore size, 8 *μ*m; Costar, Corning, Inc., Corning, NY, USA) was used for cell invasion assay, and stably transfected gastric cancer cells were harvested and suspended in FBS-free DMEM medium at a density of 1 × 10^5^ cells/mL. Next, 200 *μ*L of cell suspension was added to the upper chamber, while 500 *μ*L of DMEM containing 10% FBS was added to the bottom chamber. After culturing in a cell incubator for 24 hours, the nonmigrated cells in the upper chamber were removed with a cotton swab, and the cells invaded at the bottom of the filter were fixed in 4% paraformaldehyde at room temperature for 5 minutes. After washing the upper chamber for 1 minute, the cells were stained with crystal violet and counted at a magnification of × 100 in 5 randomly selected fields of view under a phase-contrast microscope

### 2.9. RNA Extraction and Quantitative Polymerase Chain Reaction

A spin column RNA extraction kit (Beyotime Biotechnology, Shanghai, China) was used to isolate total RNA from the cultured GC cells; then, the HiScript® RT Kit (Vazyme Biotech Co., Ltd., Nanjing, Jiangsu, China) was used according to the manual, and 1 *μ*g of total RNA was reverse transcribed into cDNA. The RNA concentration was measured by using the NanoDrop™ spectrophotometer (Thermo Fisher Scientific, Inc., Waltham, MA, USA). Quantitative polymerase chain reaction (qPCR) was performed on the ABI StepOnePlus™ real-time (RT) PCR system (Thermo Fisher Scientific, Inc., Waltham, MA, USA) with ChamQ™ SYBR (Vazyme Biotech Co., Ltd., Nanjing, Jiangsu, China).

The primer sequences used were *GAPDH*, 5′-GGAGCGAGATCCCTCCAAAAT-3′ (forward), 5′-GGCTGTTGTCATACTTCTCATGG-3′ (reverse); *BATF2*, 5′-CACCAGCAGCACGAGTCTC-3′ (forward), 5′-TGTGCGAGGCAAACAGGAG-3′ (reverse); *HDGF*, 5′-CTCTTCCCTTACGAGGAATCCA-3′ (forward), 5′-CCTTGACAGTAGGGTTGTTCTC-3′ (reverse); *METTL3*, 5′-TTGTCTCCAACCTTCCGTAGT-3′ (forward), 5′-CCAGATCAGAGAGGTGGTGTAG-3′ (reverse); and *PPP2CA*, 5′-CAAAAGAATCCAACGTGCAAGAG-3′ (forward), 5′-CGTTCACGGTAACGAACCTT-3′ (reverse).

### 2.10. Western Blot Analysis

The lysate buffer of the protease inhibitor mixture and the 1% phosphatase inhibitor mixture were added to gastric cancer cells and lysed on ice for 10 minutes to extract the proteins. Protein concentration of each group was quantitatively detected by protein quantitative kit (BCA method, Beyotime Biotechnology, Shanghai, China). Before the WB experiment, protein lysates were added into the 5× SDS-PAGE protein loading buffer in proportion, boiled for 10 minutes, and stored in the refrigerator at -20°C. Protein extract (30-50 *μ*g) was extracted for precast gel electrophoresis. After electrophoresis, the PVDF membrane (Beyotime Biotechnology, Shanghai, China) was transferred and then sealed with 5% skimmed milk powder at room temperature for 1 hour. After slight rinsing of the blocking solution with TBST, the diluted primary antibody was incubated overnight at 4°C, and the next day with the corresponding diluted secondary antibody was incubated at room temperature for 1 h. Finally, an appropriate amount of developer solution was added in a dark room before exposure strips were performed. The primary antibodies used in this study were PP2A-C*α*/*β* (1 : 500; Santa Cruz Biotechnology, Dallas, TX, USA); METTL3 (1 : 1000; Proteintech Group, Chicago, IL, USA); GAPDH (1 : 1000; Proteintech Group, Chicago, IL, USA); and *β*-actin (1 : 1000; Proteintech Group, Chicago, IL, USA).

### 2.11. Nude Mouse Tumor Formation Experiment

This animal experiment ethics is approved by Experimental Animal Center of Nanjing Medical University, approval number: IACUC-2103060. In order to establish the xenograft model of gastric cancer cells, nude mice were obtained from the animal center of Nanjing Medical University (BALA/c; 4 weeks old). Before injection, the mice were reared in a specific pathogen-free environment for 1 week. A total of 5 × 10^6^ stably transfected GC cells in 150 *μ*L of phosphate-buffered saline were injected into the flank of nude mice (*n* = 5 per group). After 3 weeks, we euthanized nude mice for the measurement of tumor volume and tumor weights.

### 2.12. Statistical Analysis

*T*-tests were used to analyze the statistical differences between normally distributed data, and *P* < 0.05 was considered statistically significant. SPSS 13.0 software (IBM Inc., Armonk, New York, USA) was used for statistical analysis.

## 3. Results

### 3.1. PP2Ac*α* and METTL3 Are Both Abnormally Expressed in Gastric Cancer Tissue and Related to Gastric Cancer Prognosis

**Figure 1:**
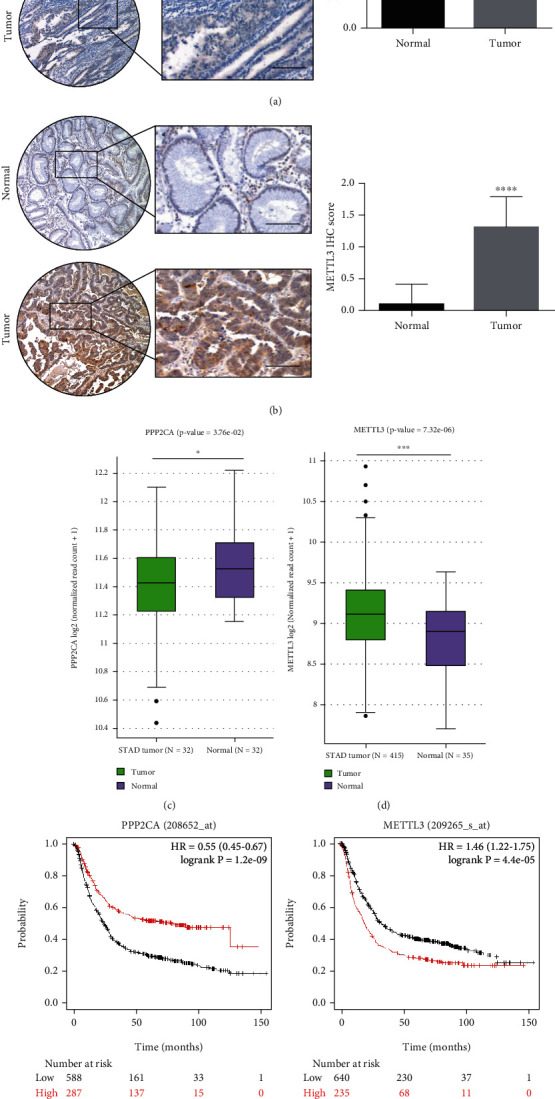
*PPP2CA* and *METTL3* expression levels in GC and relationships with GC prognosis. (a, b) Representative pictures of PP2Ac*α* and METTL3 levels in GC tissue and normal gastric mucosal tissue adjacent to the cancer (magnification, ×40 and ×100; scale bars = 200 *μ*m). (c, d) *PPP2CA* and *METTL3* mRNA levels in The Cancer Genome Atlas database (*P* = 3.76*e* − 02 and *P* = 7.32*e* − 06, respectively; *P* < 0.05 is statistically significant). (e, f) In the Kaplan-Meier plotter database, the prognostic values of *PPP2CA* and *METTL3* for overall survival in GC patients were statistically significant (*P* < 0.05). The picture can be downloaded at http://kmplot.com/analysis/index.php?p=service&cancer=ovar. ^∗^*P* < 0.05, ^∗∗^*P* < 0.01, and ^∗∗∗^*P* < 0.001. GC: gastric cancer.

To study the role of PP2Ac*α* and METTL3 in the progression of GC, we performed immunohistochemistry on 10 pairs of GC tissue and normal gastric mucosal tissue adjacent to the cancer. According to the above Materials and Methods, immunohistochemistry scoring was performed based on the scoring standard, and the results showed that the level of PP2Ac*α* in GC tissue was significantly lower than that in normal gastric mucosal tissue adjacent to the cancer (*P* < 0.001; Figure 1(a)), while the level of METTL3 was significantly increased in GC tissue (*P* < 0.0001; Figure 1(b)). The Cancer Genome Atlas (TCGA) database (https://www.cancer.gov) was used to compare the expression levels of the *PPP2CA* and *METTL3* genes in GC tissue and normal gastric mucosal tissue adjacent to the cancer. *PPP2CA* expression was significantly decreased in GC tissue (32 tumor samples vs. 32 normal samples; *P* = 3.76*e* − 02; Figure 1(c)), while *METTL3* expression was increased significantly in GC tissue (415 tumor samples vs. 35 normal samples; *P* = 7.32*e* − 06; Figure 1(d)). The results of prognostic analysis through the Kaplan-Meier plotter website (http://kmplot.com/analysis/) showed that the prognoses of the high *PPP2CA* expression and low *METTL3* expression groups were significantly better than those of the respective control groups (*P* = 1.2*e* − 09 and *P* = 4.4*e* − 05, respectively; Figures 1(e) and 1(f)). The above results suggested that PP2Ac*α* levels were significantly decreased and METTL3 levels were significantly increased in GC tissue. In addition, both the *PPP2CA* and *METTL3* genes were closely related to GC prognosis. The different roles of *PPP2CA* and *METTL3* in the progression and prognosis of GC require further exploration.

### 3.2. Inhibition of PP2Ac*α* Results in Higher METTL3 Protein Levels

**Figure 2:**
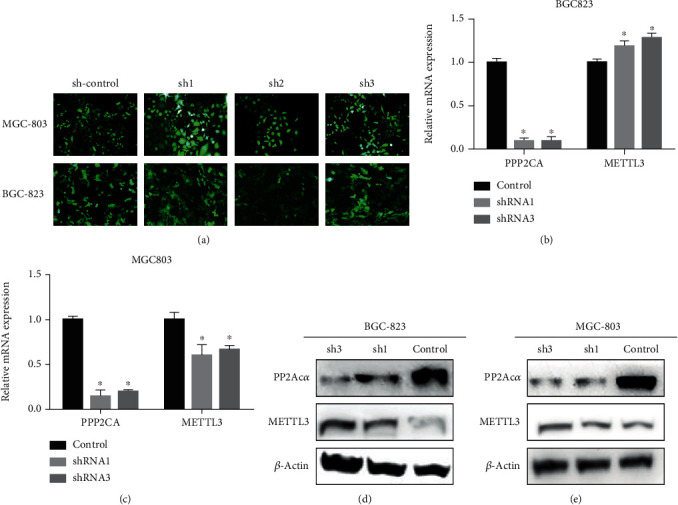
Effect of PP2Ac*α* inhibition on METTL3 levels. (a) The *PPP2CA* gene was knocked down in gastric cancer cells (BGC823 and MGC803) by lentivirus. (b, c) After knocking down *PPP2CA*, RT-qPCR was used to detect the expression of the *METTL3* gene in the BGC823 and MGC803 cells. (d, e) Western blot analysis was used to detect METTL3 levels in the BGC823 and MGC803 cells after PP2Ac*α* inhibition ^∗^*P* < 0.05.

To explore the relationship between PP2Ac*α* and METTL3, we used lentivirus-mediated shRNA to knock down the *PPP2CA* gene in BGC823 and MGC803 cells. After 48 hours of transfection, the control, shRNA1, and shRNA3 groups with strong fluorescent expression (Figure 2(a)) were screened for puromycin, and GC cells with stable and low levels of PP2Ac*α* were obtained. The successful knockdown at the mRNA and protein levels was verified (Figures 2(b)–2(e)), and METTL3 protein levels were significantly increased (Figures 2(d) and 2(e)). The mechanism of this wane-and-wax relationship deserves further exploration.

### 3.3. Inhibition of PP2Ac*α* Upregulates METTL3 through p-ATM

**Figure 3:**
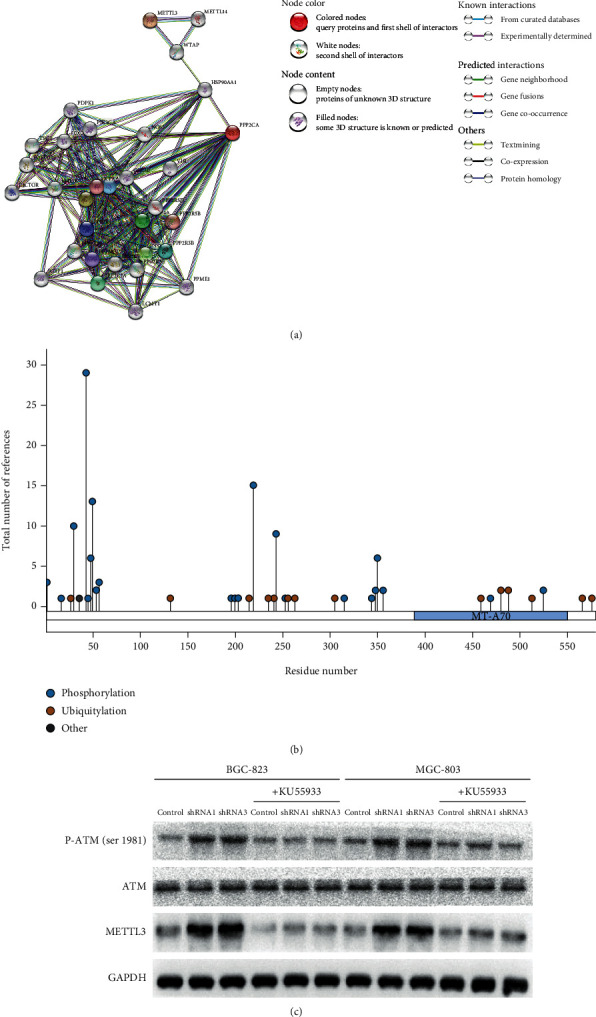
Effect of PP2Ac*α* inhibition on METTL3 levels. (a) The protein interaction network between PP2Ac*α* and METTL3 was queried through the STRING website. (b) The PhosphoSite website was used to query the phosphorylated modification of the amino acid sequence of METTL3. (c) Western blot analysis was used to detect changes in METTL3, ATM, and p-ATM levels before and after adding KU55933.

According to the above results, we first used the Protein-Protein Interactions website to analyze the relationship between PP2Ac*α* and METTL3. We found that PP2Ac*α* and METTL3 establish links via the middle proteins WTAP and HSP90AA1 (Figure 3(a)). However, we could not clarify which one was the upstream or downstream protein. Therefore, we needed to find another molecular mechanism to identify links between PP2Ac*α* and METTL3. PP2Ac*α* plays the key role in maintaining phosphorylation homeostasis. We thus used the PhosphoSite website (https://www.phosphosite.org) to search the amino acid sequences of the phosphorylation sites of METTL3, and we found that METTL3 was rich in phosphorylation (Figure 3(b)), suggesting that the levels or functions of METTL3 may be regulated by PP2Ac*α*. Based on this conjecture, through the PubMed database, we found that the phosphate groups of METTL3 could be added by the ataxia-telangiectasia mutated (ATM) kinase. The phosphate groups added onto the serine or threonine of this kinase can be removed by PP2A [21, 22]. In addition, PP2Ac*α* inhibition can upregulate p-ATM levels, which can enhance ATM kinase activity [23]. In sum, we proposed that PP2Ac*α* inhibition could upregulate METTL3 levels by enhancing the kinase activity of ATM. To test this speculation, we added KU55933, which is an ATM kinase inhibitor, to the medium of the GC cells. After 48 hours, we extracted the proteins of the GC cells to conduct a western blot assay. The experimental results showed that the p-ATM and METTL3 levels were all decreased compared to before treatment, while the total ATM level did not change significantly (Figure 3(c)). In conclusion, PP2Ac*α* inhibition was found to upregulate METTL3 levels by stimulating the kinase activity of ATM in GC cells. Some experiments have shown that high METTL3 levels are closely related to the malignant progression of GC [12, 13]. Therefore, we used phenotypic experiments to detect the malignant phenotype of GC cells after inhibiting PP2Ac*α* before and after adding KU55933.

### 3.4. Inhibition of PP2Ac*α* Promotes the Malignant Phenotype of GC Cells In Vitro

**Figure 4:**
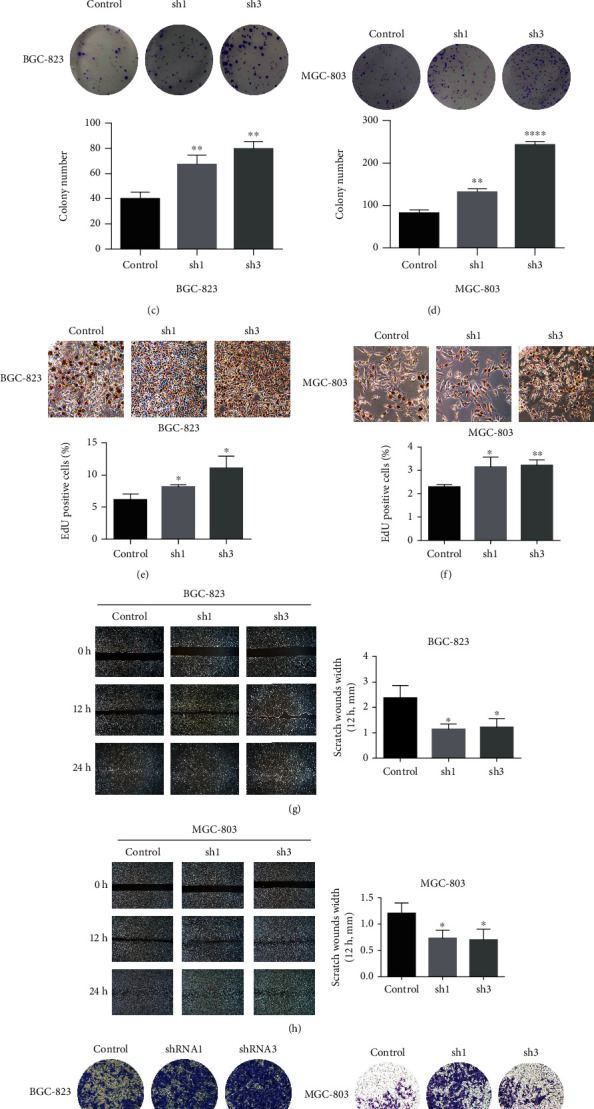
Effect of PP2Ac*α* inhibition on the proliferation, migration, and invasion of gastric cancer cells in vitro. Cell Counting Kit-8 (a, b), clone formation (c, d), and EdU (e, f) tests were used to detect the effect of *PPP2CA* knockdown on the proliferation of BGC823 and MGC803 cells. (g, h) The scratch test was used to detect the effect of *PPP2CA* knockdown on the migration of BGC823 and MGC803 cells. (i, j) The Transwell experiment was used to detect the effect of *PPP2CA* knockdown on the invasion of BGC823 and MGC803 cells. ^∗^*P* < 0.05, ^∗∗^*P* < 0.01, ^∗∗∗^*P* < 0.001, and ^∗∗∗∗^*P* < 0.0001. EdU: 5-ethynyl-2′-deoxyuridine.

To explore the effect of PP2Ac*α* inhibition on GC cells, various phenotypic experiments were carried out in vitro. The results of the CCK8 experiment showed that on the 4th and 5th days, the proliferation of the experimental group was significantly greater than that of the control group (*P* < 0.05; Figures 4(a) and 4(b)). Similarly, in the clone formation experiment (Figures 4(c) and 4(d)) and EdU cell proliferation detection experiment (Figures 4(e) and 4(f)), significantly greater proliferation ability was found in the sh1 and sh3 groups compared to the control group (*P* < 0.05). GC cells in the ibidi chamber were paved at the same density (5 × 10^5^ cells/mL), and the healing of scratches at 0, 12, and 24 hours after adhesion was observed. It was found that the healing ability of the shRNA1 and shRNA3 groups was significantly faster than that of the control group at 12 hours (Figures 4(g) and 4(h)). The Transwell invasion experiment showed that after 24 hours of culture, the invasion ability of the experimental group was significantly stronger than that of the control group (*P* < 0.01; Figures 4(i) and 4(j)). The above phenotypic experiments proved that PP2Ac*α* inhibition could promote the proliferation, migration, and invasion of GC cells.

### 3.5. Inhibition of PP2Ac*α* Promotes GC Cell Proliferation In Vivo

**Figure 5:**
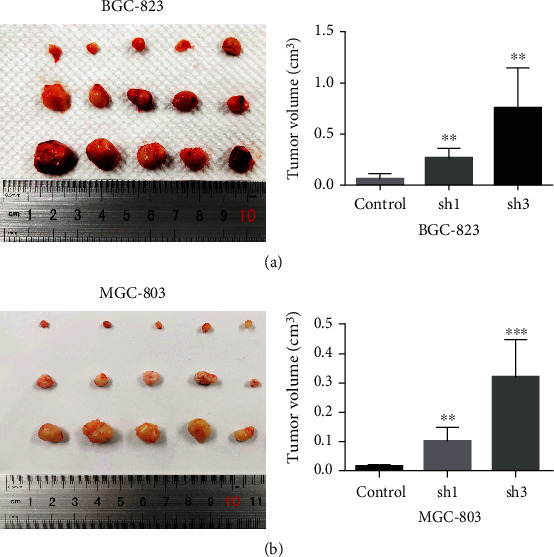
Effect of PP2Ac*α* inhibition on the proliferation of gastric cancer cells in vivo. (a, b) BGC823 and MGC803 cells transfected with LV-sh-*PPP2CA-*1,3 or LV-sh-NC were subcutaneously injected into nude mice (*n* = 5 per group), and then, 4 weeks after injection, the tumors were removed, and tumor volumes were measured. LV: lentivirus; NC: normal control.

Stable knockout cells (MGC803/LV-sh*PPP2CA* and MKN28/LV-sh*PPP2CA*) and their corresponding control cells were subcutaneously inoculated into the axillae of 4-week-old male nude mice (*n* = 5 per group). After 4 weeks, all mice were euthanized, and the tumors were isolated and removed. The results showed that PP2Ac*α* inhibition significantly promoted the tumorigenicity of GC cells in the sh1 and sh3 groups in vivo. Compared with the control group, the tumor volume increased significantly in the experimental group (*P* < 0.01; Figures 5(a) and 5(b)). These data further confirmed that knockdown of the *PPP2CA* gene could promote GC cell proliferation in animal models.

### 3.6. Inhibition of ATM Kinase Activity Can Reverse the Malignant Progression of Gastric Cancer Cells That Is Promoted by Inhibiting PP2Ac*α*

**Figure 6:**
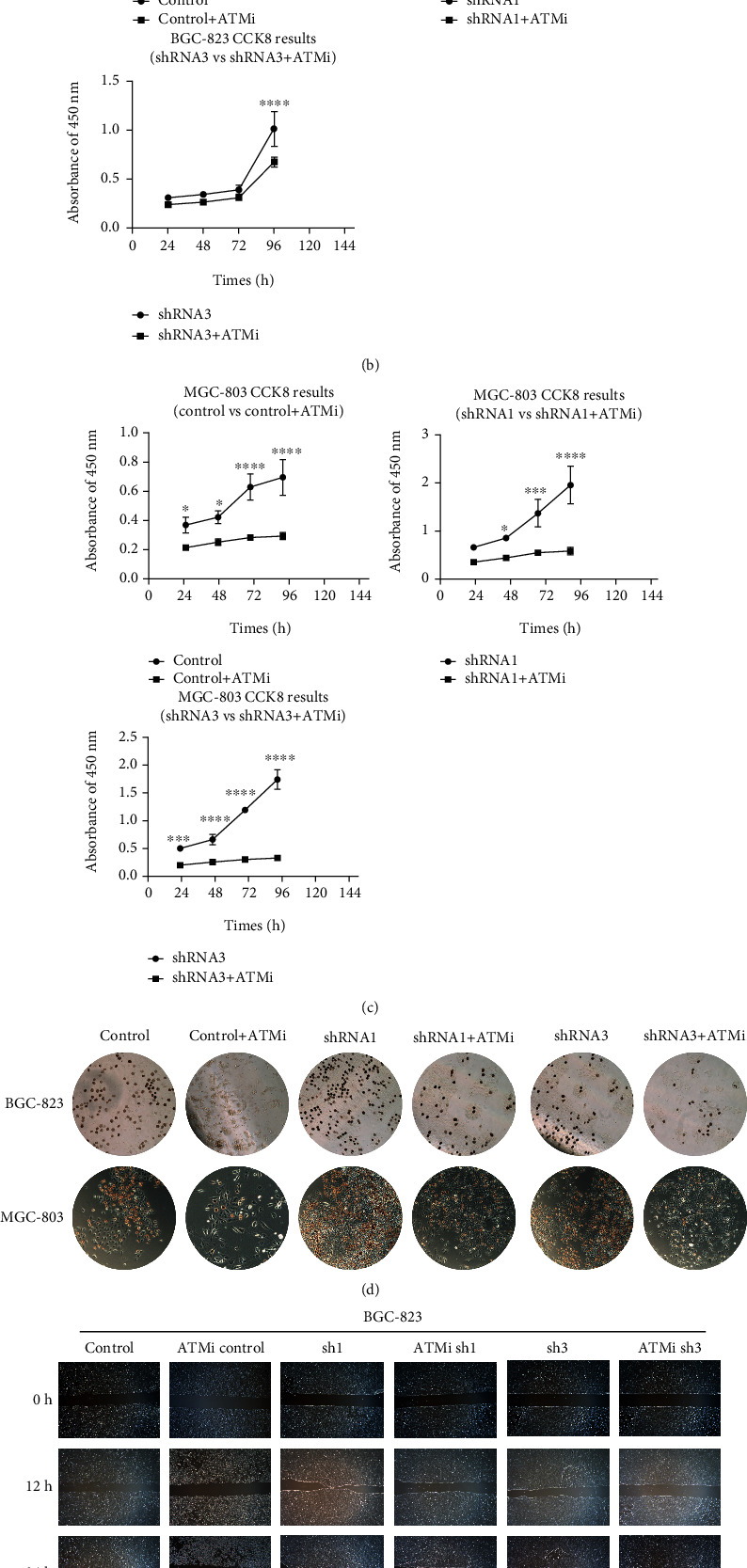
Effect of the inhibition of ATM activity on the malignant phenotype of gastric cancer cells. (a) After ATM activity was inhibited, the morphological changes of BGC-823 and MGC-803 cells were microscopically observed (magnification, ×10). (b, c) After ATM activity was inhibited, the cell proliferation of the BGC-823 and MGC-803 groups was detected by using the Cell Counting Kit-8. (d) After ATM activity was inhibited, the cell proliferation of the BGC-823 and MGC-803 groups was microscopically observed following the EdU cell proliferation test. (e, f) The migration of BGC-823 and MGC-803 cells was detected by using the scratch test. ^∗^*P* < 0.05, ^∗∗^*P* < 0.01, ^∗∗∗^*P* < 0.001, and ^∗∗∗∗^*P* < 0.0001. EdU: 5-ethynyl-2′-deoxyuridine.

After KU55933 was used to inhibit the ATM activity of the GC cells, the morphological changes of the BGC-823 and MGC-803 cells were observed, and the proliferation and migration abilities of the GC cells were detected. We found that the apoptosis of the GC cells in each BGC-823 group was increased, and the epithelial-mesenchymal transition characteristics were weakened (Figure 6(a)). That is, the GC cells became round, and the looseness between cells decreased. The CCK-8 and EdU cell proliferation assay results showed that the proliferation abilities of the BGC-823 and MGC-803 cells were significantly inhibited (Figures 6(b)–6(d)). The scratch test results showed that the migration ability of the GC cells was significantly inhibited (Figures 6(e) and 6(f)). These results suggest that the inhibition of ATM activity can reverse the enhancement of the malignant phenotype of GC cells that is induced by inhibiting PP2Ac*α*. It can be concluded that PP2Ac*α* inhibition upregulates METTL3 levels by stimulating the kinase activity of ATM, thereby promoting the malignant phenotype of GC cells.

## 4. Discussion

The poor prognosis associated with advanced GC has become a major public health problem [24–26]. The radical resection of GC has been well-developed since the 1980s, chemotherapy regimens have improved in recent years [24, 27, 28], and advanced intervention methods, such as arterial interventional embolization for distant metastases and intraperitoneal hyperthermic perfusion therapy, have emerged. However, the prognoses of patients with advanced GC have not reached public expectations. Nevertheless, in recent years, it has been discovered that molecular targeted drugs can significantly prolong the survival of patients with malignant tumors [29, 30], which is promising when it comes to curing GC. Combined with the ongoing breakthrough in the research of targeted tumor therapy [31], the molecular mechanism of GC is worthy of in-depth research in order to improve the therapeutic targets for GC and lay the foundation for better diagnosis and treatment of GC in the future.

To explore the molecular mechanism of advanced GC, this study used the most extensively modified proteins in the body as entry points. PP2A and m6A are the important components of phosphorylation homeostasis maintenance and RNA methylation modification, respectively. Between them, PP2A has been favored by researchers due to the complexity of its trimer structure, especially the regulatory subunit B, with its substrate specificity and functional diversity, which enriches the functions of the PP2A holoenzyme [8, 32]. However, the implementation of PP2A's functional diversity is inseparable from its core enzyme, which is composed of the structural subunit A and catalytic subunit C [33]. As an important part of the PP2A core enzyme, PP2Ac*α* is highly conservative, and PP2Ac*α* dysfunction often leads to the loss of PP2A holoenzyme activity, leading to a variety of life activity disorders in the body, in turn inducing various diseases [34]. So far, relevant basic research on GC has not involved PP2Ac*α*/*PPP2CA*. The TCGA and Kaplan-Meier plotter databases show that low *PPP2CA* expression is related to the poor prognosis of GC. Therefore, studying the expression imbalance of *PPP2CA* is crucial for the in-depth exploration of the pathogenesis of GC. M6A has become a hot research topic in recent years due to its dynamic and reversible methylation modification characteristics, and it has been found to have more and more important roles in various diseases [9, 35–38]. METTL3 is the core of m6A modification, and changes in the levels or methylation function of METTL3 have been found in the progress of many diseases, so METTL3 function and level abnormalities are often important research points. The correlation research of METTL3 in GC is not exceptional, and most results have shown that increased METTL3 levels promote the progression of GC [11, 39–41].

This study found that PP2Ac*α* inhibition significantly upregulated METTL3 protein levels. However, through the PubMed database, we did not find a correlation study to explain the connection between PP2Ac*α* and METTL3. Using the STRING database to query the interaction network between PP2Ac*α* and METTL3, we did not find a superior or subordinate regulatory relationship between them. Through the PhosphoSite website, we found that METTL3 has a large number of phosphorylation sites in the amino acid sequence, suggesting that METTL3 can be affected by kinase phosphorylation modification, and phosphorylation modification is often accompanied by changes in protein levels and functions.

A previous study found that ATM kinase phosphorylates the serine 43 (S43) site in the amino acid sequence of METTL3 to upregulate the level and function of METTL3. The activated METTL3 locates the position of DNA double-strand breaks (DSBs). The DSB-related RNA is methylated, and then, the m6A recognition protein YTHDC1 recognizes this methylation and recruits the RAD51 and BRCA1 proteins to perform homologous recombination repair on the damage to maintain a stable genome. Therefore, cells with low METTL3 levels lack effective homologous recombination repair, which increases the instability of the genome and leads to cell death. Tumor cells with high METTL3 levels are more likely to respond to DSBs, stabilize their own genome, and maintain their malignant phenotype and drug resistance [21]. In particular, the activity of ATM, as the upstream kinase of METTL3, can be regulated by PP2Ac*α*. Experiments have shown that PP2Ac*α* inhibition, which leads to the upregulation of the autophosphorylation of the ATM Ser1981 site, activates the activity of ATM [23].

In summary, it is speculated that in GC cells, inhibiting PP2Ac*α* can upregulate the activity of ATM, leading to the phosphorylation of the S43 position of the METTL3 amino acid sequence, which may activate the METTL3 methylation function and upregulate the protein level of METTL3, ultimately enhancing the malignant phenotype of GC cells. To verify this speculation, we extracted GC cell proteins to perform western blot experiments. The results showed that PP2Ac*α* inhibition led to increased p-ATM (Ser1981) and METTL3 levels, while the total ATM protein level did not change significantly. These results verify that the inhibition of PP2Ac*α* upregulates the activity of the ATM kinase in GC cells and is accompanied by high METTL3 levels.

Subsequently, we used KU55933 to inhibit the activity of ATM in each group of GC cells in rescue experiments. The western blot analysis results showed that after inhibiting ATM activity, the METTL3 protein levels decreased significantly. This result clarified the regulatory relationship between p-ATM and METTL3. In addition, through cell phenotyping experiments to compare the malignant phenotype of GC cells before and after adding KU55933, we found that PP2Ac*α* inhibition promoted the malignant phenotype of GC cells, but this could be reversed by adding KU55933.

It can be concluded that PP2Ac*α* inhibition promotes increased METTL3 levels by upregulating ATM activity, and it ultimately enhances the malignant phenotype of GC cells. PP2Ac*α* is the upstream of this signal axis, and its expression imbalance is the root cause of the activation of this axis. Combined with the upregulation of *PPP2CA* expression, it will inhibit the malignant phenotype of malignant tumor cells such as colon cancer, thyroid cancer, and prostate cancer. Targeted therapy of PP2Ac*α* may help to control the malignant progression of gastric cancer.

Of course, this study has limitations. Our understanding of the regulation of downstream targets by METTL3 still needs to be supplemented by follow-up studies. However, it is undeniable that this study has enriched the molecular mechanism research related to GC and laid the foundation for basic follow-up research of the clinical diagnosis and treatment of GC.

## Data Availability

The shRNA sequences used in this article were obtained from the following website: https://www.sigmaaldrich.com/life-science/functional-genomics-and-rnai/sirna/mission-predesigned-sirna.html.
